# Biosensor for Multimodal Characterization of an Essential ABC Transporter for Next-Generation Antibiotic Research

**DOI:** 10.1021/acsami.2c21556

**Published:** 2023-03-03

**Authors:** Karan Bali, Charlotte Guffick, Reece McCoy, Zixuan Lu, Clemens F. Kaminski, Ioanna Mela, Róisín M. Owens, Hendrik W. van Veen

**Affiliations:** † Department of Chemical Engineering and Biotechnology, University of Cambridge, CB3 0AS Cambridge, U. K.; ‡ Department of Pharmacology, University of Cambridge, CB2 1PD Cambridge, U. K.

**Keywords:** atomic force microscopy, biosensor, electrochemical impedance spectroscopy, electrophysiology, MsbA, supported lipid bilayer, PEDOT:PSS, structured illumination microscopy

## Abstract

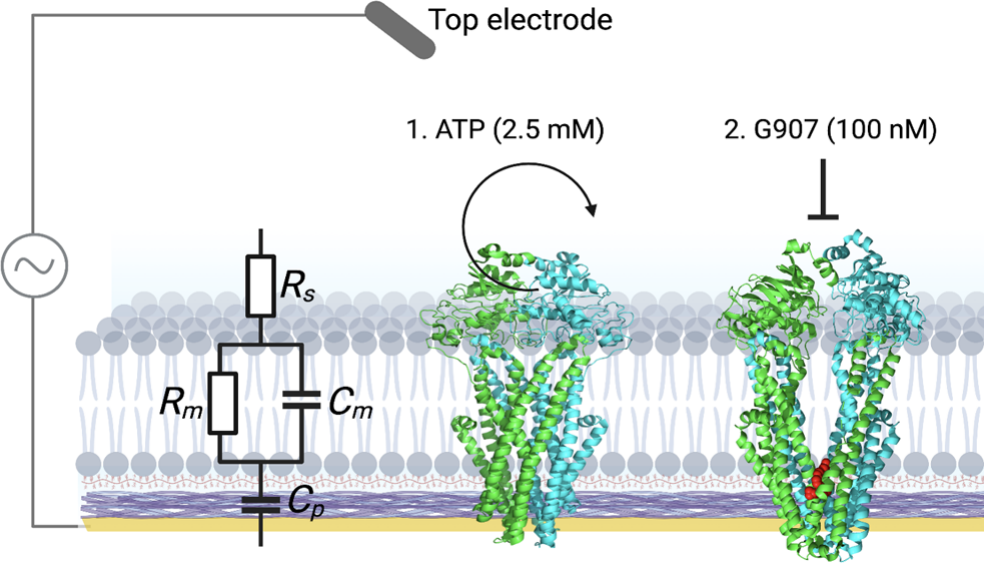

As the threat of antibiotic resistance increases, there is a particular focus on developing antimicrobials against pathogenic bacteria whose multidrug resistance is especially entrenched and concerning. One such target for novel antimicrobials is the ATP-binding cassette (ABC) transporter MsbA that is present in the plasma membrane of Gram-negative pathogenic bacteria where it is fundamental to the survival of these bacteria. Supported lipid bilayers (SLBs) are useful in monitoring membrane protein structure and function since they can be integrated with a variety of optical, biochemical, and electrochemical techniques. Here, we form SLBs containing *Escherichia coli* MsbA and use atomic force microscopy (AFM) and structured illumination microscopy (SIM) as high-resolution microscopy techniques to study the integrity of the SLBs and incorporated MsbA proteins. We then integrate these SLBs on microelectrode arrays (MEA) based on the conducting polymer poly(3,4-ethylenedioxy-thiophene) poly(styrene sulfonate) (PEDOT:PSS) using electrochemical impedance spectroscopy (EIS) to monitor ion flow through MsbA proteins in response to ATP hydrolysis. These EIS measurements can be correlated with the biochemical detection of MsbA-ATPase activity. To show the potential of this SLB approach, we observe not only the activity of wild-type MsbA but also the activity of two previously characterized mutants along with quinoline-based MsbA inhibitor G907 to show that EIS systems can detect changes in ABC transporter activity. Our work combines a multitude of techniques to thoroughly investigate MsbA in lipid bilayers as well as the effects of potential inhibitors of this protein. We envisage that this platform will facilitate the development of next-generation antimicrobials that inhibit MsbA or other essential membrane transporters in microorganisms.

## Introduction

The ESKAPE pathogens (*Enterococcus faecium*, *Staphylococcus aureus*, *Klebsiella pneumoniae*, *Acinetobacter baumannii*, *Pseudomonas aeruginosa*, and *Enterobacter* species) are a public health priority by the World Health Organization in the fight against antibiotic resistance. They provide important drug targets in our quest to develop next-generation antibiotics.^1^ One of these targets is the ATP-binding cassette (ABC) transporter MsbA in the plasma membrane of Gram-negative ESKAPE bacteria and in *Escherichia coli*, *Salmonella typhimurium*, *Vibrio cholerae*, and others.^2−5^ MsbA plays an essential role in the viability and survival of these bacteria by mediating the translocation of core-Lipid-A and glycerophospholipids across the plasma membrane.^6−9^ In further steps at the outer leaflet of the plasma membrane, a polysaccharide moiety (O-antigen) is ligated to core Lipid-A to form full-length lipopolysaccharides (LPS) which, together with phospholipids, are essential for forming the protective and rigid outer membrane that make Gram-negative bacteria so impervious to antibiotics.^10,11^ Furthermore, as MsbA has been experimentally more accessible than some of its mammalian homologues over the past two decades, it has also been studied as a model system, enabling advancements in our understanding of protein structures and transport mechanisms in the ABC superfamily.

Functionally active homodimeric MsbA consists of two transmembrane domains (TMDs), each made up of six transmembrane helices (TMHs) that form the substrate translocation pathway, a pair of highly conserved cytosolic nucleotide-binding domains (NBDs) that bind and hydrolyze ATP to drive the transport reaction, and the intracellular domains (ICDs) that join the TMDs to the NBDs (Figure S1). In the transport reaction, MsbA follows an alternating access mechanism where an inward-facing conformation allows for the binding of substrate by diffusion from the cytoplasm and inner leaflet of the plasma membrane,^12−14^ while an ATP-bound outward-facing conformation enables the release of the substrate in the outer leaflet of the membrane and cellular exterior. The subsequent hydrolysis of the nucleotide allows MsbA to return to the inward-facing conformation. For certain substrates, including cytotoxic ethidium, erythromycin, and chloramphenicol, and the phospholipid phosphatidylethanolamine, transport by MsbA is stimulated by the input of a chemical proton gradient (interior alkaline) in addition to nucleotide binding and hydrolysis.^9,15^ Along with the wildtype MsbA (MsbA-WT) form, our study includes two well-established mutant forms of the protein. The MsbA-ΔK382 mutant is an ATPase deficient mutant while the MsbA-TripRA mutant is unable to transport Lipid-A and PE across the membrane.^9^

To date, two classes of compounds have been reported as inhibitors of MsbA, a series of quinoline-based molecules that prevent proper NBD closure to suppress ATP hydrolysis and substrate transport^16,17^ and tetrahydrobenzothiophene-based molecules that force a collapsed inward-facing state, thereby disrupting the NBD-TMD communication and yielding higher basal ATPase activities.^18,19^ Although promising, the development of new, precise drug-screening methods is required to advance these antimicrobials and further novel drug classes for clinical applications. Supported lipid bilayers (SLBs) recreate the environment of a cell membrane in an in vitro setting. SLBs made from proteoliposomes are extremely effective as a tool to test the functions of lipids and membrane proteins. The most common method for forming SLBs is based on the fusion and rupture of (proteo)liposomes on a solid support, thus forming a cell membrane mimic. The SLB platform lends itself to analyses by a range of techniques, such as atomic force and fluorescence microscopy, which help to uncover the structural features of membrane proteins in the context of the lipid environment while simultaneously gaining structural information on the lipid bilayer itself.^20,21^ Drug-screening studies are also possible as seen in the example of antibiotic screening with clinically relevant bacterial membranes.^22^ In the context of precise monitoring of membrane protein activity, a particularly exciting new area of research is the integration of SLBs with bioelectronic devices.

Bioelectronics is a growing field of research that aims to combine biological systems with electronic monitoring. The emergence of organic semiconductors has allowed for the development of organic bioelectronic devices that show great promise due to their potential low manufacturing costs, biocompatibility, and inherent ion-to-electronic signal amplification.^23^ The active material of an organic bioelectronic device is the conducting polymer which, owing to its mixed conductivity (both ionic and electronic), can transduce events in a biological system into an electronic output. A widely used conducting polymer is poly(3,4-ethylenedioxy-thiophene) poly(styrene sulfonate) (PEDOT:PSS).^24^ By coating microelectrode arrays (MEA) with PEDOT:PSS, it is possible to create devices that can be integrated with SLBs and report on their biological properties with a number of advantages compared to existing systems. PEDOT:PSS is able to enhance the sensitivity by reducing the impedance of the device compared to uncoated metal electrodes.^25^ In addition to this, the “cushioned” nature of the material means that it is biocompatible (i.e., it does not denature membrane proteins) while the transparent nature of the material allows for optical monitoring of the integrated biological system.^26^ By monitoring the electrical properties of SLBs using electrochemical impedance spectroscopy (EIS) with these devices, a variety of biological phenomena have been monitored. For instance, studies have shown that PEDOT:PSS-coated electrodes can be used to monitor the opening and closing of ion channels, the disruption of membranes by antibiotics, and the fusion of virus particles with cell membranes.^27−29^ Here, we show that EIS, combined with optical and biochemical techniques, provides a novel method for monitoring ATP-dependent MsbA activity as well as characterizing the efficacy of inhibitors that block this activity.

## Results and Discussion

**Figure 1:**
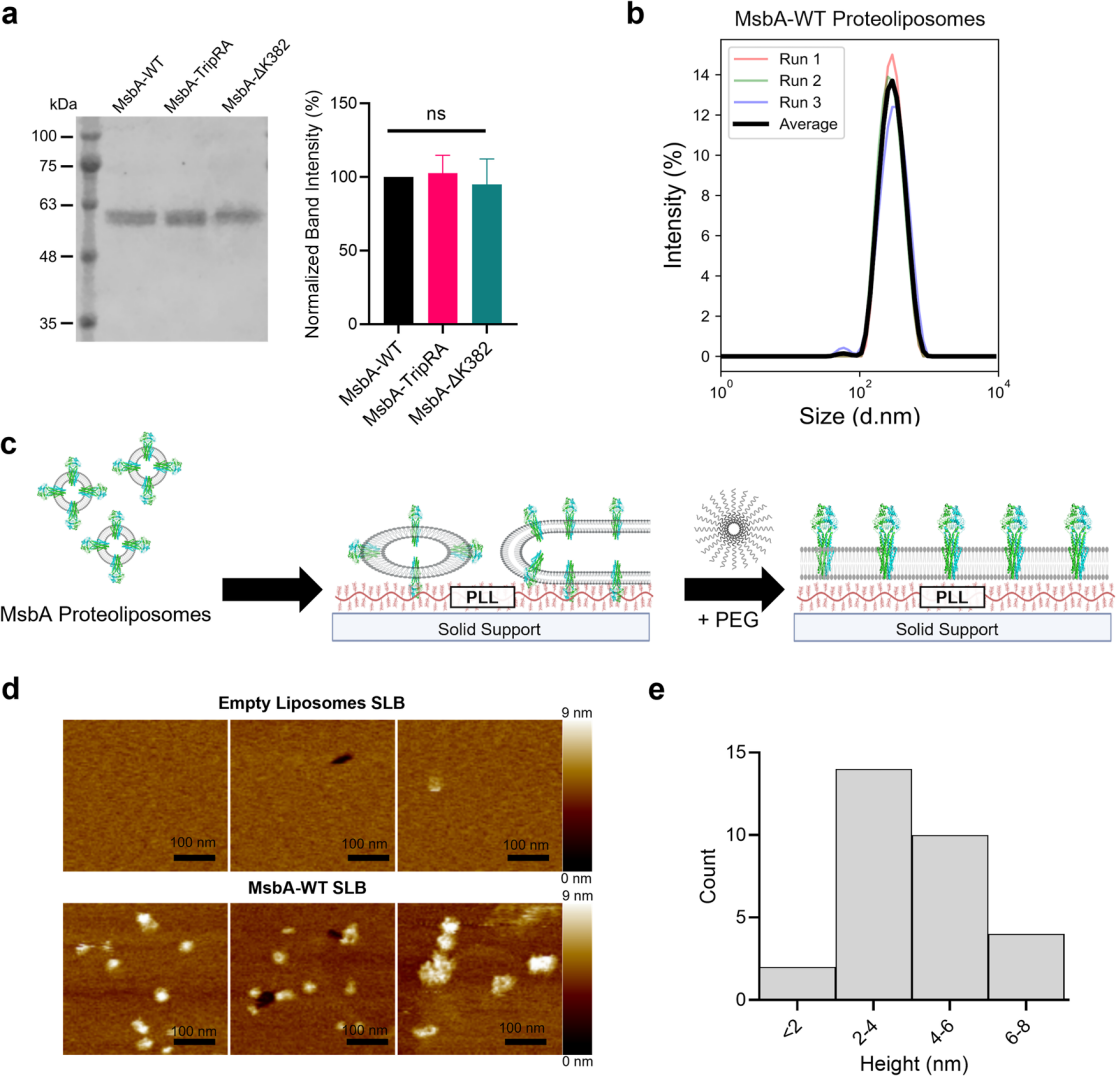
Characterization of MsbA-containing proteoliposomes and SLBs. (a) Coomassie staining of proteoliposome samples on SDS-PAGE (left) (∼800 μg lipid per lane) demonstrated equal incorporation of MsbA-WT, MsbA-TripRA, and MsbA-ΔK382 proteins in the proteoliposomes. Molecular mass markers are indicated on the left. Histogram (right) shows mean band intensity from three separate preparations relative to MsbA-WT, error bars represent s.e.m. (b) Size of MsbA-WT-containing proteoliposomes determined by DLS. The mean hydrodynamic size is 269.6 ± 4.2 nm with a polydispersity index of 0.18 ± 0.03 (error represents s.d. (*n* = 3)). (c) Schematic of the fusion of MsbA-containing proteoliposomes and polyethylene glycol (PEG)-assisted rupture on a poly-l-lysine (PLL)-coated solid support. (d) AFM analyses of MsbA-WT-containing SLBs (bottom) and SLBs prepared from empty liposomes (top) on mica. Images represent three enlarged sections of a 2 × 2 μm AFM scan of the two SLBs in Figure S2. (e) Range of particle heights, taken from the AFM images for MsbA-WT bilayers in (d), show the distribution of MsbA particle size protruding from the lipid bilayer.

To use MsbA in EIS measurements, the affinity-purified protein was reconstituted in a native bilayer-mimicking environment as previously described for biochemical studies.^15,30^ Specifically, *E. coli* MsbA with an NH_2_-terminal His_6_-tag was expressed in the Gram-positive bacterium *Lactococcus lactis* that lacks LPS, Lipid-A, and endogenous MsbA. Following purification into detergent solution, MsbA was reconstituted in liposomes prepared with polar *E. coli* lipid extract and egg-yolk PC (3:1 w/w). Different from isolated plasma membrane vesicles, these proteoliposomes lack cytoplasmic constituents and alternative primary-active and secondary-active membrane transporters, thus allowing direct observations of the ATP hydrolysis and transport activity of MsbA.^15^ The reconstitution of wildtype MsbA (MsbA-WT) or MsbA mutants (namely the MsbA-TripRA and MsbA-ΔK382 forms discussed in the Introduction section) into liposomes was evident by the presence of a ∼60 kDa signal on Coomassie-stained SDS-PAGE (Figure 1a), which is absent in empty liposomes.^15^ The particle size distribution of the proteoliposomes was examined using dynamic light scattering (DLS). The proteoliposome suspensions gave monodisperse peaks at 269.6 ± 4.2 nm with a polydispersity index of 0.18 ± 0.03 (Figure 1b), indicating the absence of aggregates that could interfere with SLB formation.

To form SLBs using these proteoliposomes, we employed the liposome fusion technique as shown in Figure 1c. Briefly, the proteoliposomes are added to a solid substrate functionalized with poly-l-lysine (PLL). This is because the presence of phospholipids with negatively charged phosphate groups in our polar lipid extract cause the (proteo)liposomal membrane surface to have an overall negative charge, so the positively charged PLL aids with the liposome binding to the surface. The proteoliposomes fuse and rupture to form an SLB. This process was stimulated by a washing step using 30% (w/v) polyethylene glycol 8000 (PEG8k), which facilitated the osmotic disruption of any remaining intact proteoliposomes.^31^ Atomic force microscopy (AFM) imaging of MsbA-containing SLBs on mica provided further structural information on the lipid bilayers and on protein incorporation and orientation (Figures 1d and S2). Comparison of the bilayer heights of the MsbA-containing SLBs and empty SLBs, prepared with empty liposomes in the same method, produced comparable heights of 3.35 ± 0.34 and 3.56 ± 0.21 nm, respectively. Additionally, MsbA-containing SLBs showed distinct protrusions that were not present in empty SLBs. With a mean height of 4.34 ± 1.59 nm above the height of the SLBs (Figure 1e), these protrusions fall well within the height range (maximum height of ∼6.5 nm) of the NBDs plus ICDs (Figure S1) as suggested by cryo-EM studies of MsbA dimers in lipidic nanodiscs.^32^ These AFM analyses demonstrate the presence of approx. 25 MsbA transporters per μm^2^ of lipid bilayer in the SLBs with their NBDs exposed to the external buffer (“NBD-out”). As the height of the periplasmic loops that connect the TMHs in MsbA lie within the error margin of height measurements of the lipid bilayer, it is not possible to detect proteins by AFM that insert with their NBDs exposed to the interior of the SLBs (“NBD-in”). During the reconstitution of purified MsbA into proteoliposomes, MsbA inserts in a unidirectional inside-out fashion.^15^ If the SLBs form from the proteoliposomes via the “parachute” model of liposome rupture,^26^ this would lead to the dominance of the “NBD-out” form in the SLBs as observed in AFM analyses. Only the “NBD-out” configuration can be activated by the addition of Mg-ATP in the external buffer.

**Figure 2:**
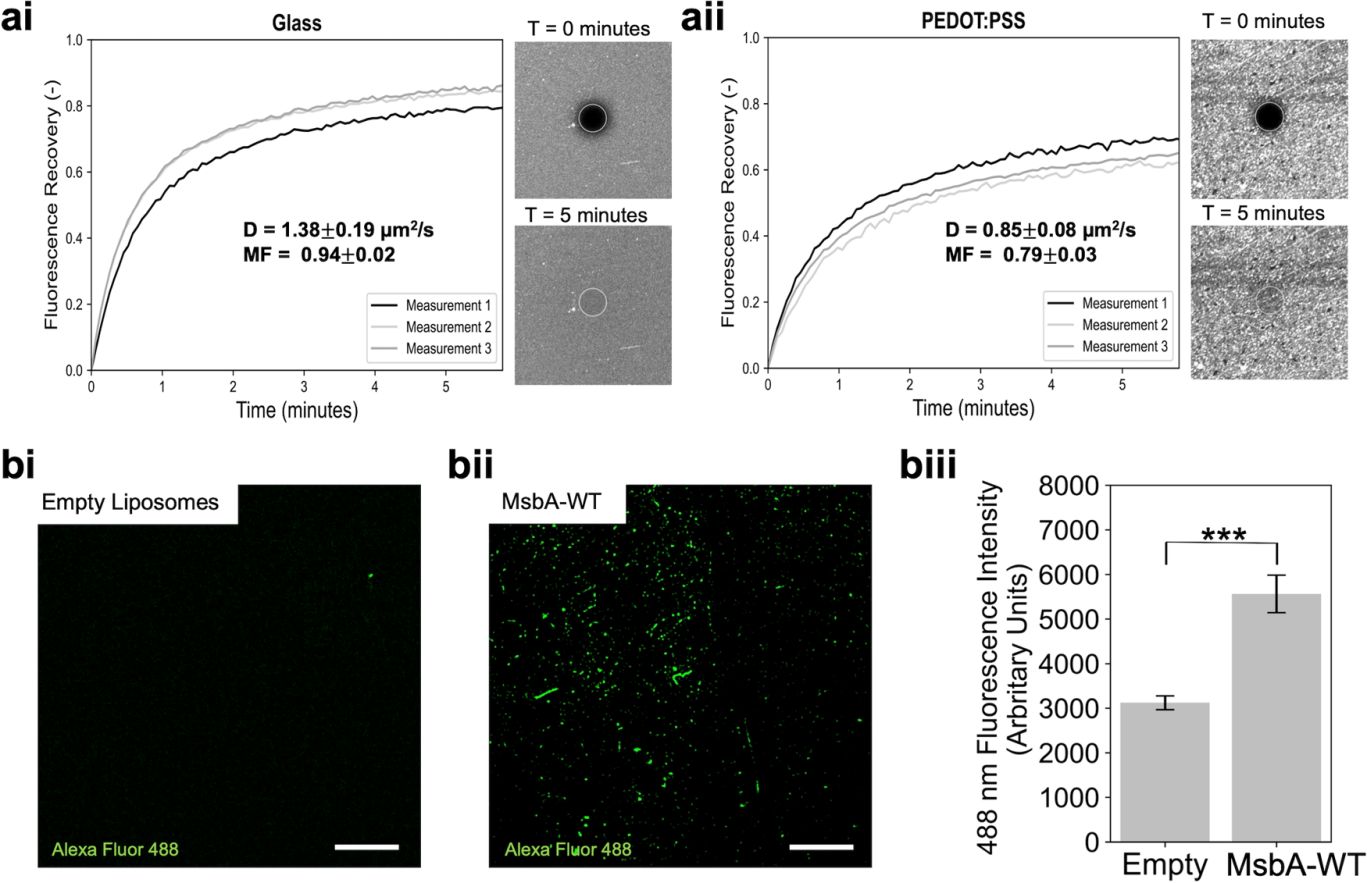
FRAP and SIM characterization of MsbA SLBs. (ai, ii) FRAP data for the SLBs on glass (ai) and PEDOT:PSS (aii). For both, the fluorescence in the bleached circle (diameter 30 μm) recovered over time. The calculated diffusion coefficient (*D*) and mobile fraction (MF) values were 1.39 ± 0.19 μm^2^/s and 0.94 ± 0.02, respectively (ai), and 0.85 ± 0.08 μm^2^/s and 0.79 ± 0.03, respectively (aii). Data were collected by bleaching three separate areas on the same bilayer. Error represents s.d. of the three measurements. (bi-iii) SIM imaging for empty SLBs (bi) and MsbA-containing SLBs (bii) on PEDOT:PSS. MsbA was detected with antiHis tag primary antibody and Alexa Fluor 488 secondary antibody. (biii) Bar chart showing the difference in 488 nm fluorescence between the two types of bilayers. Data were collected by measuring three areas from three separate images each. Error bars represent the standard error of the mean; two-way analysis of variance was conducted (***P ≤ 0.001). Scale bars are 5 μm.

A commonly used technique to ascertain the mobility and contiguous nature of a lipid bilayer is fluorescence recovery after photobleaching (FRAP).^33^ We first stained the MsbA-WT proteoliposomes with the fluorescent dye rhodamine-18 (R18) and formed SLBs on glass using the PEG-assisted liposome fusion method (Figure 2ai). Then, a 30 μm spot in the bilayer was photobleached and the recovery of fluorescence in this area was monitored over time. The diffusion coefficient (*D*), which measures the rate of lipid movement in the bilayer, was 1.39 ± 0.19 μm^2^/s. The mobile fraction (MF), which measures the fraction of lipids that are mobile in the SLB, was 0.94 ± 0.02. Both values show that the SLB is highly mobile and agree with previous values recorded for R18 stained lipid bilayers formed on glass.^33^ Since PEDOT:PSS is the conducting polymer substrate in our EIS measurements, it was important to ascertain that contiguous and mobile SLBs could also be formed on this substrate. Therefore, we generated MsbA-WT-containing SLBs on PLL-functionalized PEDOT:PSS-coated glass slides and measured FRAP (Figure 2aii). The *D* and MF values were 0.85 ± 0.08 μm^2^/s and 0.79 ± 0.03, respectively, which are comparable to published values for SLBs on PEDOT:PSS.^26^ The lower values compared to those obtained with lipid bilayers on glass are expected and are presumably due to the added surface roughness of PEDOT:PSS that reduces the mobility of the SLB. The added roughness of PEDOT:PSS compared to glass may also explain the features present in the images of SLBs here, likely aggregates of PEDOT:PSS or unruptured proteoliposomes. We also formed SLBs on glass and PEDOT:PSS using empty liposomes (Figure S3) and showed fluorescence recovery over time with *D* values (1.22 ± 0.09 and 1.31 ± 0.13 μm^2^/s for glass and PEDOT:PSS, respectively) higher than those observed for MsbA SLBs, reflecting the smoother nature of these SLBs and, thus, the faster diffusion of lipids. The data demonstrate that the SLBs do form on the conducting polymer surface, allowing us to leverage the properties of the material, notably its electrical and biocompatible properties, to conduct electrochemical characterization of MsbA protein activity in the SLB.

Following these characterizations, the presence of purified MsbA in SLBs on glass slides (Figure S4) and PEDOT:PSS-coated slides (Figure S5) was detected by immunostaining with an antiHis antibody and a fluorescent Alexa Fluor 488-labeled secondary antibody imaged using structured illumination microscopy (SIM). The NH_2_-terminal His_6_-tag of MsbA lies at the cytoplasmic extension of TMH 1 and, once in SLBs, should be accessible to external buffer systems. Comparing the MsbA-containing SLBs to empty SLBs, the fluorescence in the Alexa Fluor 488 channel increased in the presence of MsbA (Figure 2bi–iii). From these data, we can conclude that our methods generate mobile bilayers on PEDOT:PSS, which contain MsbA protein in an NBD-out orientation.

**Figure 3:**
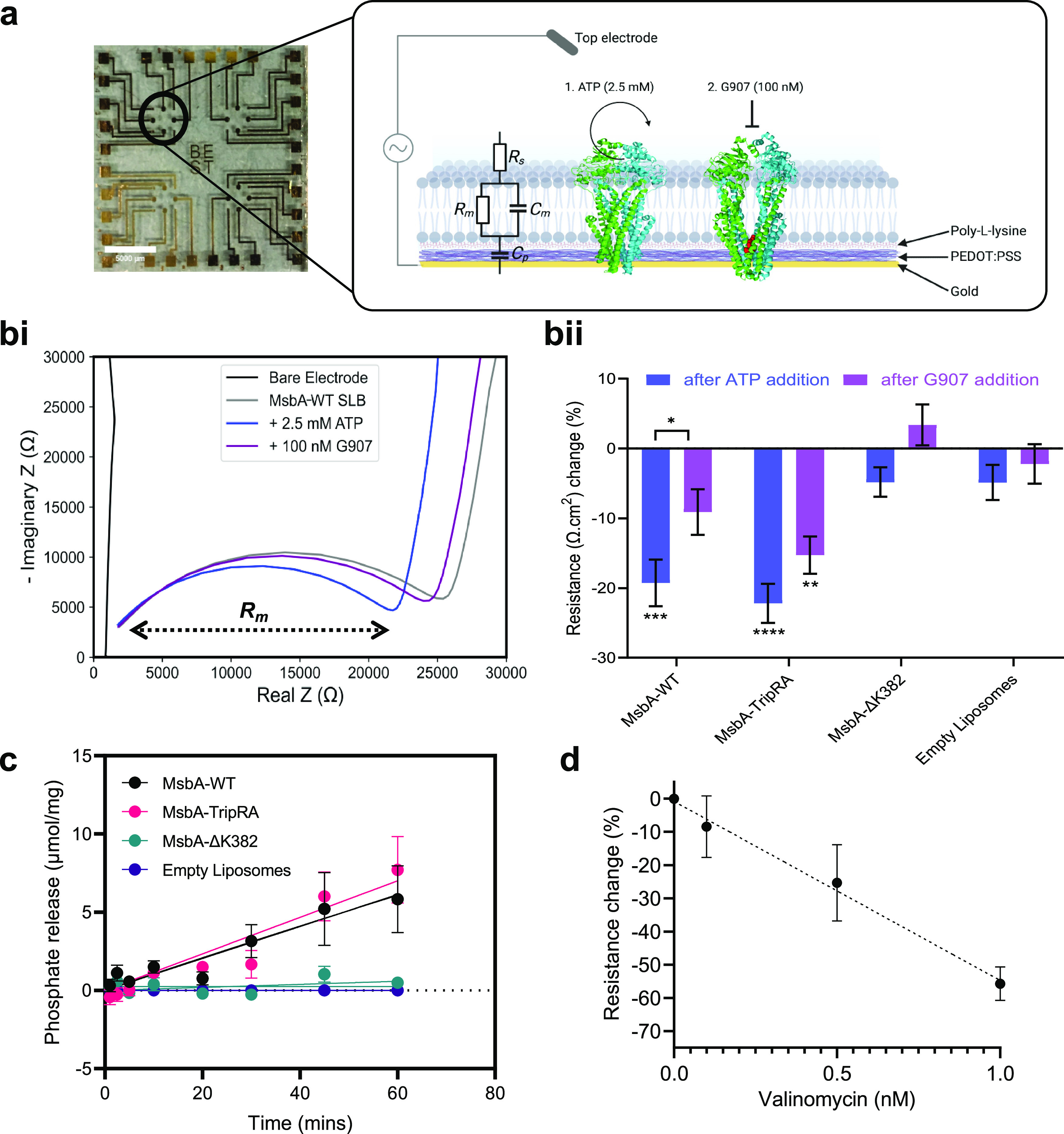
Electrical and biochemical measurements for MsbA-containing SLBs. (a) Image of a PEDOT:PSS-coated MEA, consisting of four arrays with eight electrodes in each array. Scale bar is 5 μm. The black circle indicates a single array with the inset depicting a schematic of the MsbA SLB (PDB: 5TTP, 6BPL) formed on an electrode. The equivalent circuit used to model the SLB and extract membrane resistance values is shown on the bilayer to the right. (bi) EIS measurement represented as a Nyquist plot (real vs imaginary impedance) for a representative electrode and MsbA-WT SLB. Measurements are taken for the PEDOT:PSS electrode (i.e., with no SLB present) after SLB formation and after the subsequent additions of 2.5 mM ATP and 100 nM G907. The width of the semicircle portion of the graph is used to calculate the membrane resistance (*R*_m_). (bii) Resistance change (measured as the normalized resistance change versus SLB resistance) following the successive addition of ATP (blue) and G907 (purple) for MsbA-WT or MsbA-TripRA-containing SLBs in comparison to SLBs containing the ATPase-inactive MsbA-ΔK382 mutant or without MsbA (empty SLBs). Asterisks indicate significance relative to empty liposomes in the same buffer condition. The bracket represents the significance of the with-inhibitor versus without-inhibitor comparison for the same protein (two-way analysis of variance; *P ≤ 0.05; **P ≤ 0.01; ***P ≤ 0.001; ****P ≤ 0.0001). (c) ATPase activity of MsbA proteins in SLBs prepared on PEDOT:PSS at pH 6.8 measured as the release of phosphate over time. MsbA-WT (black) and MsbA-TripRA (red) showed a basal activity of 100 ± 13 and 117 ± 12 nmol Pi/min/mg, respectively, with mg of protein estimated from AFM data. MsbA-ΔK382 (green) and empty liposomes (purple) showed limited phosphate release. (d) Calibration curve of valinomycin concentration against increased ion permeation created by measuring the % resistance change relative to the SLB resistance in the absence of valinomycin. All data points and error bars represent mean ± s.e.m. observations in three experiments (*n* = 3) with independently prepared batches of liposomes that were used to form the SLBs.

Confident that MsbA-containing SLBs are formed on PEDOT:PSS, the electrical characterization of MsbA proteins was performed using EIS on PEDOT:PSS-coated MEA (Figure 3a). EIS can be used to measure the properties of a biological system by applying a sinusoidal alternating voltage (AV) to the sample, which is known to stabilize the membrane resistance and transmembrane current noise for SLBs on silicon surfaces,^34^ and by measuring the complex impedance *Z* over a frequency range where *Z* is a sum of the real part (e.g., resistance) and imaginary part (e.g., capacitance) of the impedance.^35^ EIS data can be represented by a Bode plot, which plots the magnitude of *Z* and phase angle against frequency, and by a Nyquist plot where the imaginary and real components of *Z* are plotted against each other for each frequency. By fitting an equivalent circuit to the data, it is possible to extract quantitative electrical properties of the system, such as resistance and capacitance.^36^ For the MsbA-containing SLBs integrated on an MEA, we used a well-established equivalent circuit to fit the data where the PEDOT:PSS polymer is represented as a capacitor, the electrolyte as a resistor, and the SLB as a resistor and capacitor in parallel (Figure 3a).^37^ When investigating the properties of an SLB integrated with MEA using EIS, the Nyquist plot is a useful way of representing the data where the width of the semicircle portion of the graph is used to calculate the resistance of the lipid bilayer.

Using the MsbA-WT-containing SLBs in our devices, we were able to observe the characteristic semicircle shape on the Nyquist plot (Figure 3bi). By fitting an equivalent circuit to model the data, we extracted a membrane resistance of 216 ± 74 Ω cm^2^, which was consistent with previously reported resistance values for membranes formed with synthetic lipids.^27^ Due to the electrode-to-electrode variation in membrane resistance, we measured normalized resistance change relative to baseline (before nucleotide addition) for each specific electrode to understand the effect of ATP addition in the MsbA-containing SLB system. The addition of 2.5 mM ATP to the buffer system, enabling the rate of ATP hydrolysis to approach Vmax, resulted in a 19.2 ± 3.3% decrease in membrane resistance, indicating an increase in ion permeation through the SLB (Figure 3bii). The formation of SLBs from MsbA-containing proteoliposomes on our devices allows for direct measurements of ATP-dependent changes in MsbA in a native-like membrane environment containing *E. coli* lipids in the absence of other membrane proteins or cytosolic proteins that are part of the *E. coli* proteome.

To confirm that the observed response in MsbA-containing SLBs is linked to ATP binding and hydrolysis by MsbA, 100 nM ATP hydrolysis and transport inhibitor G907^9,16^ was introduced into the system. By wedging into a conserved transmembrane pocket, G907 and related quinoline compounds trap MsbA in an inward-facing conformation. The second allosteric mechanism of antagonism occurs through structural and functional uncoupling of the NBDs.^16^ When G907 was introduced to our system, the membrane resistance of ATP-hydrolyzing MsbA-containing SLBs increased by 10.0 ± 3.3%. When identical experiments were performed with empty SLBs, we obtained a similar initial membrane resistance (200 ± 48 Ω.cm^2^) as observed for MsbA-containing SLBs. However, the addition of ATP and G907 to the empty SLBs did not cause significant changes in the membrane resistance (Figure 3bii). Furthermore, when the experiments were repeated with the MsbA-ΔK382 mutant, which incorporates equally well as MsbA-WT in the proteoliposomes (Figure 1a) but only shows residual 6–10% ATPase activity of MsbA-WT due to the absence of the catalytic Walker A lysine residue,^38^ the shifts in membrane resistance were not significantly different from those observed in empty SLBs (Figure 3bii). A representative Bode and Nyquist plot for each condition, as well as tables of the raw resistance values, is shown in the Supporting Information (Figure S6, Table S1).

The hydrolysis of ATP by the MsbA proteins in the SLBs on PEDOT:PSS was confirmed in colorimetric malachite green assays which report the release of Pi in this reaction. In the presence of 2.5 mM ATP, MsbA-WT exhibited an activity of 0.013 ± 0.004 nmol Pi/min (Figure 3c). Using AFM images to estimate the amount of protein in the SLBs, this equates to 100 ± 13 nmol Pi/min/mg equivalent to the ATPase rates obtained for suspended proteoliposomes of the same lipid composition.^9^ In the case of the MsbA-ΔK382 SLBs, an ATPase activity of 10 ± 4 nmol Pi/min/mg was obtained, which is ∼10% of the MsbA-WT. From these data, we conclude that the ATP-dependent resistance changes in the MsbA-containing SLBs require ATP binding and subsequent hydrolysis by MsbA. The inhibitory effect of G907 on the resistance changes is particularly important since it shows that the system is well suited for detecting the effects of small molecule inhibitors that could be further developed as next-generation antibiotics targeting MsbA.

As the negatively charged PSS chain in PEDOT:PSS interacts with alkali metal cations such as Na^+^ and K^+^ for signal generation,^39^ the ATP hydrolysis-dependent shift in the membrane resistance in MsbA-containing SLBs can potentially be attributed to the movement of K^+^ as the dominant cation in our buffer system across the membrane into the PEDOT:PSS polymer.^40^ To further characterize this shift, we tested the electrical response to transbilayer K^+^ flow induced by the ionophore valinomycin, which shows K^+^-selective uniport across bilayers. The addition of stepwise increasing concentrations of valinomycin to empty liposome SLBs was associated with a proportional reduction in the membrane resistance (Figure 3d). Using linear regression, the % change related to the ATP hydrolysis-associated shift for MsbA-WT corresponded to an approximate valinomycin concentration of 0.343 nM. Assuming a complete partitioning of valinomycin in the phospholipid bilayer, this would correspond to a valinomycin:MsbA ratio of 33:1 (see Materials and Methods). With reported turnover numbers for valinomycin in phospholipid bilayers of up to 2000 K^+^/s,^41,42^ the ATP-dependent shift in membrane resistance for MsbA might, therefore, correspond to K^+^ transport rates of up to 6.6 × 10^4^/s per MsbA transporter. This rate is high compared to the turnover number for ATP hydrolysis by MsbA of approx. 1/s to 10/s, which reflects the alternating access of the central substrate-binding chamber, and is more in line with the reported passing of 10^4^–10^7^ ions/s through the open pore of ion channels.^43^ Repetition of the EIS measurements with MsbA-WT-containing SLBs in Na^+^ buffers resulted in identical responses as for K^+^ buffers (Figure S7), suggesting that MsbA is nonselective for these monovalent cations. Given that MsbA is a multidrug and lipid ABC transporter,^9^ it is noteworthy that K^+^/Na^+^ currents were previously observed in the MsbA orthologue LmrA from *L. lactis*(44,45) and ABC multidrug transporter PDR5 from yeast,^46^ and that K^+^ transport has also been observed for the ABC multidrug transporter YbhFSR from *E. coli*(47) and the major facilitator superfamily multidrug transporters MdfA from *E. coli*(48) and MdrP from *Planococcus maritimus*.^49^ Furthermore, a link exists between ion conductance and lipid transport in lipid scramblases. For example, mammalian TMEM16F is functional as a Ca^2+^-activated phosphatidylserine channel, which is almost equally permeable to Li^+^, Na^+^, K^+^, Rb^+^, and Cs^+^, but much more permeable to Ca^2+^and Ba^2+^. The pore is also permeable to cations as large as *N*-methyl-d-glucamine^+^ and tetraethylammonium^+^.^50^

Interestingly, we observed an ATP-dependent decrease of 22.2 ± 2.8% in membrane resistance in SLBs containing the MsbA mutant TripRA,^9^ which is not significantly different to the data obtained with MsbA-WT (*P* = 0.8436). The substrate-binding chamber of this mutant lacks three arginine residues (R78, R148, and R296) that are critical for Lipid-A and PE transport.^9^ However, consistent with previous comparisons of ethidium transport by MsbA-WT and MsbA-TripRA in cells, the ethidium^+^ transport activity in proteoliposomes near Vmax conditions (Figure S8), as well as the ATPase activity of the TripRA mutant (117 ± 12 nmol Pi/min/mg), were not significantly (*P* = 0.5509) different from the observations for WT protein (100 ± 13 nmol Pi/min/mg). Therefore, the EIS measurements, which are nonselective for K^+^, Na^+^, and perhaps other cations, appear to be associated with ethidium transport rather than lipid flipping by MsbA. It should be noted that K^+^ and Na^+^ are not the only ions in our buffer system; HEPES^+^ and NH_4_^+^ (as a K^+^ analogue) might also be translocated to produce decreases in membrane resistance. We also cannot exclude that MsbA-WT and MsbA-TripRA might exhibit a similar ATP-dependent transport activity for lipids other than Lipid-A and PE in our SLBs, and that, during the EIS measurements, this activity reduces the surface area of the lipid bilayer in contact with the PEDOT:PSS polymer, thereby exposing K^+^/Na^+^ binding sites in the polymer to ions in the buffer and creating the signal response.

## Conclusions

In this study, we have shown a multiparametric approach to investigate the ABC transporter MsbA in SLBs. In particular, we bring together optical, biochemical, and electrical measurements to probe the activity of MsbA and various mutant forms of the transporter. Our data suggest that the ATPase activity of MsbA is coupled to an increased cation permeation through the membrane. The exact contributions of cation and lipid transport by MsbA to signal generation in the EIS measurements are still unknown, and further work will focus on discerning the detailed characteristics of this permeation reaction. The benefit of using the MEA system to perform EIS measurements is the increased sensitivity to ion movement for membrane transporters that typically have low catalytic activities and rates of ion conductance compared to ion channels. For the first time, the signals generated in the EIS measurements have been correlated with both biochemical assays as well as an ion flux calibration curve using valinomycin. Furthermore, the MEA system provides a quantitative readout of the blocking effect of the drug G907, thus showing the compatibility of our devices with ABC transporters and the potential of the system for antimicrobial drug development. Therefore, our multiparametric approach provides a robust platform for drug-screening and mechanistic studies with MsbA and other ABC transporters of therapeutic interest.

## Materials and Methods

### Bacterial Strains, Cell Growth, and Protein Expression

The drug-hypersensitive *L. lactis* strain NZ9000 Δ*lmrA* Δ*lmrCD*^51^ was used for the expression of (i) wild-type MsbA (MsbA-WT), (ii) MsbA-ΔK382 with low ATPase activity, and (iii) the triple arginine MsbA mutant: R78A R148A R296A (MsbA-TripRA) with low Lipid-A and PE flipping activity^9^ from pNZ8048-derived plasmids under a nisin-inducible promotor.^52^ Overnight cultures of the lactococcal cells were grown in M17 medium (Formedium) supplemented with 25 mM glucose and 5 μg mL^–1^ chloramphenicol. Overnight cultures were diluted 1:25 (v/v) into fresh identical media and allowed to grow at 30 °C to a OD_660_ of 0.55–0.6, after which 10 pg mL^–1^ nisin-A was added to induce protein expression for 1 h.^53^

### Preparation of Inside-Out Membrane Vesicles

Lactococcal cells from 2 L cultures were harvested post protein expression at 4 °C by centrifugation (13,000 × *g*, 10 min). The cell pellet was washed once (100 mM K-HEPES, pH 7.0) and resuspended in 40 mL of the same buffer. Chicken egg white lysozyme (3 mg mL^–1^, Sigma-Aldrich) was added together with a tablet of Complete-Protease Inhibitor Cocktail (Sigma-Aldrich), and the mixture was incubated for 30 min at 30 °C. To lyse the cells, the mixture was passaged thrice through a cell disrupter (Basic *Z* 0.75-kW Benchtop Cell Disrupter, Constant Systems) at 20,000 p.s.i. Subsequently, 10 μg mL^–1^ DNase and 10 mM MgSO_4_ were added, and the resultant mixture was incubated for 30 min at 30 °C to digest DNA and RNA. Then, 15 mM K-EDTA (pH 7.0) was added, and the mixture was centrifuged for 40 min at 13,000 × *g* and 4 °C. The supernatant containing the membrane vesicles was transferred to a clean tube and centrifuged for 1 h at 125,000 × *g* and 4 °C. The membrane vesicle pellet was resuspended in 50 mM K-HEPES buffer (pH 7.0) containing 10% (v/v) glycerol to a total membrane protein concentration of 40–60 mg mL^–1^. The membrane vesicle suspension was stored in aliquots in liquid nitrogen.

### Purification of His-Tagged MsbA Proteins

His_6_-tagged MsbA proteins were purified by Ni^2+^-nitrilotriacetic acid (NTA) affinity chromatography.^15^ Approximately 200 μg Ni^2+^-NTA resin, with binding capacity of up to 50 mg mL^–1^ and bead size 45 and 165 μm, was pre-equilibrated by washing by centrifugation (175 × *g*, 1 min, 4 °C) five times with five resin volumes of ultrapure water and twice with five resin volumes of Wash Buffer A (50 mM K-HEPES (pH 8.0), 0.1 M NaCl, 10% (v/v) glycerol, 0.05% (w/v) n-dodecyl-β-d-maltoside (DDM), and 20 mM imidazole (pH 8.0)). Solubilization of target protein was achieved through addition of membrane vesicles at ∼5 mg mL^–1^ to solubilization buffer (50 mM K-HEPES (pH 8.0), 10% (v/v) glycerol, 0.1 M NaCl, and 1% (w/v) DDM). The mixture was gently shaken at 4 °C for 4 h before transferring to the washed Ni^2+^-NTA resin to be again shaken at 4 °C overnight. Resultant resin was transferred to 2 mL disposable Biospin chromatography columns (Bio-Rad) and washed with 20 resin volumes of Wash Buffer A and 20 resin volumes of Wash Buffer B (50 mM K-HEPES (pH 7.0), 0.1 M NaCl, 10% (v/v/) glycerol, 0.05% (w/v) DDM, and 20 mM imidazole (pH 8.0)). Bound protein was eluted with 400–500 μL of elution buffer (50 mM K-HEPES (pH 7.0), 0.1 M NaCl, 5% (v/v) glycerol, 0.05% (w/v) DDM, and 150 mM imidazole (pH 8.0)). Purified protein was kept on ice and used immediately.

### Preparation of Proteoliposomes

Liposomes (8 mg) were prepared from acetone-ether-washed total lipid extract from *E. coli* and egg yolk phosphatidylcholine (Avanti Polar Lipid Inc.) mixed at a ratio of 3:1 (w/w), respectively, in chloroform. Solvent was evaporated by gentle flushing with N_2_ gas, after which the lipid film was hydrated in liposome buffer (20 mM K-HEPES, 100 mM NH_4_SCN, 50 mM K_2_SO_4_, pH 6.8) to a concentration of 4 mg mL^–1^. Hydrated lipids were extruded 11 times through a 400 nm polycarbonate filter (Fisher) with a mini extruder (Avanti) to produce unilamellar liposomes of a homogenous size. Extruded liposomes were destabilized by adding Triton X-100 until the maximum OD_540_ has just passed. Liposomes were prepared in batches of up to 9 mL and, following extrusion, were aliquoted for the addition of each MsbA protein (for proteoliposomes) or equal volume of elution buffer (for empty liposomes). Purified protein was mixed with destabilized liposomes in a ratio of 1:50 (160 μg) and left gently shaking at RT for 30 min. Detergent was removed through successive incubations with SM-2 Bio-Beads (Bio-Rad), activated with washing three times with methanol, once with ethanol, four times with ultrapure water, and once with liposome buffer: 80 mg mL^–1^ at room temperature for 2 h, 8 mg mL^–1^ at 4 °C for 2 h, and finally 8 mg mL^–1^ at 4 °C for 18 h. For the preparation of DNA-loaded proteoliposomes, calf-thymus DNA (Trevigen) was added at 1 mg mL^–1^ prior to hydrating. Before use, DNA-loaded proteoliposomes were incubated at 30 °C for 20 min with 10 mM MgSO_4_ and 10 μg mL^–1^ DNAse I to remove any unincorporated DNA in the external environment. All proteoliposomes were harvested by centrifugation at 165,000 × *g* at 4 °C for 30 min and resuspended to ∼40 mg of lipid mL^–1^ in liposome buffer. In-gel quantification of incorporated recombinant proteins was assessed in Coomassie-stained SDS-PAGE using protein standards. Empty liposomes were prepared in identical conditions with elution buffer added in place of protein to lipid preparations.

### Characterization of Proteoliposomes Using DLS

DLS measurements were performed using a Zetasizer Nano S90 (Malvern Panalytical) configured with a 633 nm laser and a 90° scattering optic. 1 mL of sample (in which liposomes were diluted 40×) was transferred into a disposable plastic cuvette, and three runs were taken for each measurement. The intensity of the scattered light is used by the Zetasizer software to determine three main parameters: *Z* average, which is the weighted mean of the hydrodynamic diameter (in nm) of the particles, polydispersity index (PDI), which provides a measure for the heterogeneity of the particle size distribution, and count rate (kcps), which counts the number of photons detected per second and is related to the concentration and quality of the sample.

### Preparation of PEDOT:PSS Solution

PEDOT:PSS dispersion (Heraeus) was mixed with 5% (v/v) ethylene glycol (EG) and 0.5% (v/v) dodecylbenzenesulfonic acid (DBSA) in order to enhance film formation and conductivity. 1% (v/v) 3-glycidoxypropyltrimethoxysilane (GOPS), which is a polymer crosslinking agent, was then added, and the final solution was sonicated for 10 min and filtered through a 0.8 μm membrane prior to use.

### PEDOT:PSS Spin Coating

Initially, glass microscope coverslips (Academy, 22 × 40 mm, 0.16–0.19 mm thick) were cleaned with DI water before coating. In the optimized protocol, glass coverslips were sonicated in isopropanol and acetone for 15 min each before coating. The glass surface was then activated by air plasma treatment with a 2 L Femto chamber (Diener electronic GmbH) for 2 min to improve the homogeneity of PEDOT:PSS layer formation. 200 μL of PEDOT:PSS solution was then spin-coated onto the glass surface at 3000 rpm for 45 s at room temperature followed by soft baking at 90 °C for 1 min. This was followed by annealing at 130 °C for 45 min. The slides were then soaked in PBS or DI water for at least 4 h and then dried with N_2_ gas prior to use.

### Formation of SLBs

The substrate surface was first functionalized by incubation with 0.1% (w/v) PLL solution (Sigma) for 15 min. The PLL solution was washed away with deionized H_2_O before 100 μL of proteoliposomes (∼4 mg mL^–1^) were added for 20 min. In a final step, 30% (w/v) polyethylene glycol 8000 solution was added for 10 min to help with bilayer formation by inducing osmotic stress to rupture any remaining proteoliposomes.^31^

### Characterization of SLBs Using FRAP

Prior to analysis by FRAP, samples were fluorescently labeled. This was achieved by adding 1 μL of octadecyl rhodamine chloride 18 dye (R18) (Invitrogen) to 200 μL of proteoliposome suspension and sonicating for 15 min. A G25 spin column (GE Healthcare) was used to remove unbound/excess R18 by centrifugation at 3000 rpm for 3 min at room temperature. Lipid bilayer formation was then conducted using the protocol outlined above. FRAP measurements were conducted using an inverted Zeiss LSM800 confocal microscope with a 10× objective lens. A 30 μm diameter bleaching spot was made, and recovery of the fluorescence intensity of this spot was measured over time relative to a 50 μm diameter reference spot. The data were analyzed using MATLAB using the Soumpasis fit to extract the diffusion coefficient (*D*) according to the eq *D* = *r*^2^/4τ where *r* is the radius of the photobleached spot and τ is the characteristic diffusion time. FRAP was performed on SLBs on glass and on PEDOT:PSS-coated glass. PEDOT:PSS-coated glass was used rather than PEDOT:PSS-coated MEA as the MEA devices consisted of a solid gold electrode spin-coated with PEDOT:PSS and were opaque and inaccessible to FRAP.

### Immunostaining of SLBs and Imaging Using SIM

SLBs were prepared as in “Formation of SLBs” on glass microscope coverslips (Academy, 22 × 40 mm, 0.16–0.19 mm thick). The bilayers were incubated in 2% (w/v) BSA solution for 1 h as a blocking step and then rinsed thoroughly with liposome buffer. The primary antibody, anti6X His Tag antibody (Abcam) was added to the bilayers in a 1:100 dilution in 0.2% (w/v) BSA solution, and the bilayers were incubated at 4 °C overnight. The bilayers were washed thoroughly with liposome buffer and then incubated in goat antimouse IgG H&L (Alexa Fluor 488) secondary antibody (Abcam) in a 0.2% (w/v) BSA solution for 1 h. The bilayers were washed once in liposome buffer before imaging using SIM. The wavelengths used for excitation were 561 nm (OBIS 561, Coherent) for the lipid bilayers and 488 nm (iBEAM-SMART-488, Toptica) for the secondary antibody.

### AFM Measurements

AFM images were acquired in Scanasyst mode using ScanasystFluid+ probes (Bruker) with a nominal spring constant of 0.7 N m^–1^ and a resonant frequency of 150 kHz. Images were recorded at scan speeds of 1.5 Hz and tip–sample interaction forces between 200 and 300 pN. To resolve the morphology of the bilayers, 2 × 2 μm scans were generated. Height measurements on the bilayers were performed by taking cross-sections across different areas of interest using the Nanoscope analysis software (Bruker).

### ATPase Activity in SLBs

A colorimetric malachite green-based assay was used to detect Pi release from ATP hydrolysis by MsbA in SLBs. SLBs were prepared as described in “Formation of SLBs.” After successful bilayer formation, reconstituted proteins were incubated with liposome buffer supplemented with 2.5 mM ATP and 5 mM MgSO_4_ to a total volume of 0.5 mL. ATP hydrolysis was allowed to proceed at 30 °C, and 30 μL of reaction mixture was removed at given time points and stored on ice until full reaction time was completed. Malachite green-ammonium molybdate solution was prepared as previously described^51^ and filtered and then activated with a 1 in 100 dilution of Triton X-100 from a 10% (w/v) stock solution, immediately before use. Reaction mixture samples were added to 150 μL of prepared malachite green solution, and color was left to develop at RT for 5 min before addition of 40 μL 34% (w/v) citric acid to stop the color change. A_600_ was recorded after incubation for a further 25 min. A standard curve was prepared with 30 μL of known concentrations of KPi for each experiment.

### Substrate Transport in Proteoliposomes

Transport activity of reconstituted protein was followed in DNA-loaded proteoliposomes through the application of ethidium bromide. Transport was initiated by the 100-fold dilution of liposomes in 2 mL of ΔpH buffer (20 mM K-HEPES, 100 mM KSCN, pH 8.0) supplemented with 5 mM MgSO_4_ to impose an interior acidic pH gradient in 3.5 mL glass cuvettes (∼20 mg protein per mL). After 30 s of mixing, 2.5 mM ATP and 2 μM ethidium bromide were added and fluorescence followed for a further 10 min in an LS-55B luminescence spectrometer (Perkin-Elmer Life Sciences) with excitation and emission wavelengths of 500 and 580 nm with slit widths of 10 and 5 nm, respectively. Control experiments were run with proteoliposomes in the liposome buffer in the absence of ATP and with empty liposomes.

### Microelectrode Array Device Fabrication

The fabricated PEDOT:PSS microelectrodes are designed into arrays with circular electrodes with 450 μm in diameter or 200 μm by 200 μm square electrodes. To fabricate the devices, 4-inch glass wafers first were cleaned by sonication in acetone and then isopropanol for 15 min. The wafers are rinsed with DI water and baked 15 min at 150 °C. To pattern for contact tracks, a negative photoresist, AZ nLOF2035 (Microchemicals GmbH) was spun on the glass wafer with 3000 rpm for 45 s in a spin coater, type WS-650Mz-23nPPB from Laurell Technologies Corporation, and exposed with UV light using mask aligner (Karl Suss MA/BA6). The photoresist was developed in AZ 726 MIF developer (MIcroChemicals) for 28 s. Ti (5 nm)/Au (100 nm) layer as conductive tracks was deposited by e-beam evaporation on top of wafer, and the Ti–Au metal layer was lifted-off by soaking in Ni555 (Microchemicals GmbH) overnight. Prior to the deposition of 2 μm layer (sacrificial layer) of parylene C ((SCS), the wafer was soaked with 3% (v/v) A174 (3-(trimethoxysilyl)propyl methacrylate) in ethanol solution (0.1% (v/v) acetic acid in ethanol) 60 s to promote the parylene C adhesion on the wafer. An antiadhesive layer of Micro-90 in DI water (2% v/v solution) was spun (1000 rpm for 45 s), and then the second layer of 2 μm parylene C (SCS) was deposited. A layer of positive photoresist AZ 10XT (Microchemicals GmbH) was spun with 3000 rpm for 45 s and developed in AZ 726 MIF developer (MicroChemicals) for 6 min to pattern electrode areas. Reactive ion etching (Oxford 80 Plasmalab plus) opened the window for deposition of Clevios PH500 PEDOT:PSS (Heraeus). The PEDOT:PSS solution containing 5 vol % ethylene glycol, 0.26 vol % dodecylbenzenesulfonic acid (DBSA), and 1 vol % (3-glycidyloxypropyl)trimethyloxy-silane (GOPS) was spin-coated at 3000 rpm for 45 s. The sample was baked at 90 °C for 1 min, and the sacrificial layer was peeled off. Finally, the sample was put on a hot plate at 130 °C for 1 h before use.

### EIS Measurements

EIS was performed using a potentiostat (Autolab PG-STAT204) in a three-electrode configuration with Ag/AgCl and Pt electrodes being used as the reference and counter electrodes, respectively. Each PEDOT:PSS-coated gold electrode in a single array was sequentially used as the working electrode. The AC current was recorded within the frequency range 50–100,000 Hz with 10 data points per decade (equally spaced on a logarithmic scale). An AC voltage of 0.01 V and a DC voltage of 0 mV versus OCP were applied. For all experiments, liposome buffer supplemented with 5 mM MgCl_2_ was used as the electrolyte. After baseline bilayer measurements were recorded, 2.5 mM ATP and 100 nM G907 were added successively to the system and measurements recorded 5 min following the addition of each chemical. Data were collected and analyzed using NOVA 2.1.3 software (Metrohm Autolab).

### Valinomycin EIS Measurements

SLBs were formed as previously described on the same PEDOT:PSS-coated gold electrodes with the same electrode configuration used. Baseline EIS spectra were obtained for each SLB in liposome buffer followed by incubation with valinomycin (Sigma) (diluted in liposome buffer) at concentrations of 0, 0.1, 0.5, and 1 nM for 10 min. SLBs were washed to remove any excess valinomycin that was not incorporated into the bilayer, and EIS was performed in an identical fashion as described under “EIS measurements.” Data were analyzed using NOVA 2.1.3 where an equivalent circuit model was applied, and bilayer resistances extracted. To account for the time-dependent drift of the bilayer resistance, the resistance changes were scaled by the relative resistance change in the control group. Drift-adjusted mean percentage changes relative to the initial baseline bilayer resistances were calculated. For the calculation of the K^+^ transport rate associated with MsbA activity in the EIS measurements, the following parameters were used. The lipid bilayer was prepared in a well with a diameter of 8 mm, corresponding to a surface area of 5.027 × 10^7^ μm^2^ (1). With a buffer volume of 200 μL, 0.343 nM valinomycin equals to 4.131 × 10^10^ valinomycin molecules per well (2). Given the high octanol–water partition coefficient for valinomycin of 1.26 × 10^9^ (https://pubchem.ncbi.nlm.nih.gov/compound/3000706), we assume that valinomycin will almost completely partition in the phospholipid bilayer. A combination of (1) and (2) yields 822 valinomycin molecules/μm^2^ of lipid bilayer. As AFM revealed a density of 25 MsbA transporters/μm^2^ of lipid bilayer (Figure 1d), the ratio of valinomycin:MsbA transporter in the bilayer equals 822:25, or 33:1. Hence, if the EIS signal shift for valinomycin is equal to that for MsbA at this ratio, the K^+^ transport rate for MsbA will equal 33× the turnover number of valinomycin. With a turnover number of valinomycin of up to 2000/s,^41^ this corresponds to a flux of up to 6.6 × 10^4^ K^+^/s per MsbA transporter.

## Data Availability

Data that support the findings of this study have been deposited in the University of Cambridge research repository Apollo with DOI link https://doi.org/10.17863/CAM.93576 or are available from the corresponding authors upon reasonable request.

## Supporting Information Available

Schematic diagram of the MsbA dimer in inward- and outward-facing conformation; AFM images of empty SLBs and SLBs containing MsbA-WT; FRAP characterization on empty SLBs; immunofluorescence images for SLBs on glass or PEDOT:PSS; EIS measurements for the four SLB systems; EIS measurements for MsbA-WT-containing SLBs in the presence of Na^+^ rather than K^+^; effect of inhibitor G907 on ethidium transport by MsbA proteins in DNA-loaded proteoliposomes; and membrane resistance values extracted from EIS data for the four SLB systems (PDF)

## Abbreviations

ABC: ATP-binding cassette
AFM: atomic force microscopy
*D*: diffusion coefficient
EIS: electrochemical impedance spectroscopy
FRAP: fluorescence recovery after photobleaching
ICDs: intracellular domains
LPS: lipopolysaccharides
MEA: microelectrode arrays
MF: mobile fraction
NBDs: nucleotide-binding domains
PEDOT:PSS: poly(3,4-ethylenedioxy-thiophene) poly(styrene sulfonate)
PEG: polyethylene glycol
PLL: poly-l-lysine
R18: rhodamine-18
SIM: structured illumination microscopy
SLB: supported lipid bilayer
TMDs: transmembrane domains
TMHs: transmembrane helices
