# Supplementary material for: Biosensor for Multimodal Characterization of an Essential ABC Transporter for Next-Generation Antibiotic Research
Source: ACS Appl Mater Interfaces. 2023 Mar 3;15(10):12766–76. doi: 10.1021/acsami.2c21556 (PMC10020959; doi:10.1021/acsami.2c21556)
Supplement: Supplementary file 1 — am2c21556_si_001.pdf [file am2c21556_si_001.pdf]

SUPPORTING INFORMATION

A Biosensor for Multi-Modal Characterisation of an Essential ABC Transporter for Next-Generation Antibiotic Research

Karan Bali[1†], Charlotte Guffick[2†], Reece McCoy[1], Zixuan Lu[1], Clemens F. Kaminski[1], Ioanna Mela[1], Róisín M. Owens[1], Hendrik W. van Veen[2]

1. Department of Chemical Engineering and Biotechnology, University of Cambridge, CB3 0AS Cambridge, United Kingdom
2. Department of Pharmacology, University of Cambridge, CB2 1PD Cambridge, United Kingdom

[†] These authors contributed equally.

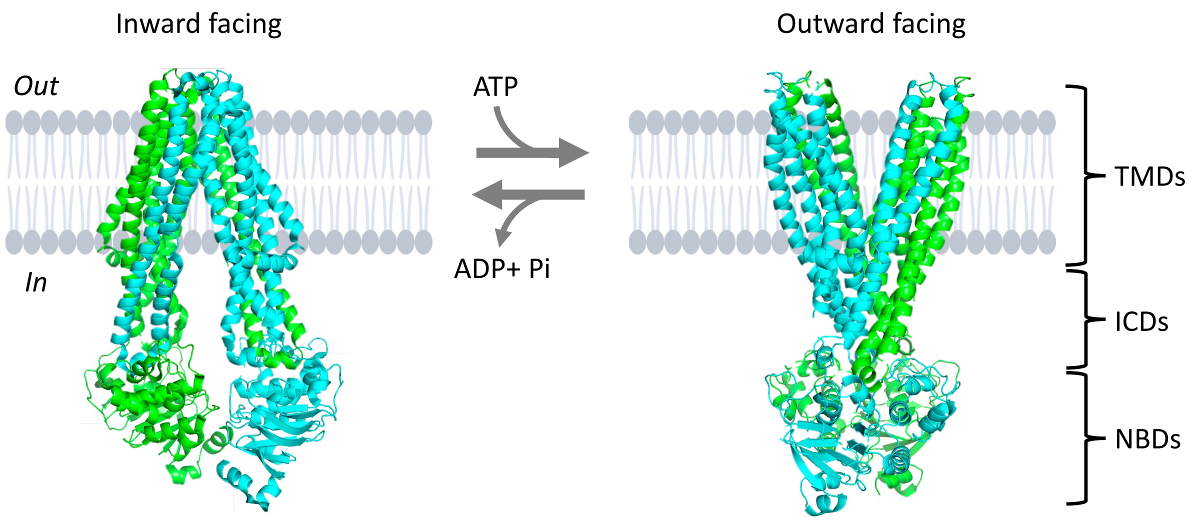

Figure S1. Schematic diagram of the conformational cycle of MsbA upon ATP binding and hydrolysis. The MsbA transporter is active as a homodimer of two half-transporters that are indicated in green and cyan. The two transmembrane domains (TMDs) each contain six transmembrane helices (TMHs). 'Inward facing' (PDB: 6BPL) and 'outward facing' (PDB: 3B60) refer to the accessibility of the central substrate binding chamber between the two TMDs. The nucleotide-binding domains (NBDs) contain conserved sequence motifs that enable the binding and hydrolysis of two ATP molecules at their interface. The Intracellular Domains (ICDs) join the TMDs to the NBDs. 'Out' and 'in' refer to the outside and inside of the plasma membrane, respectively.

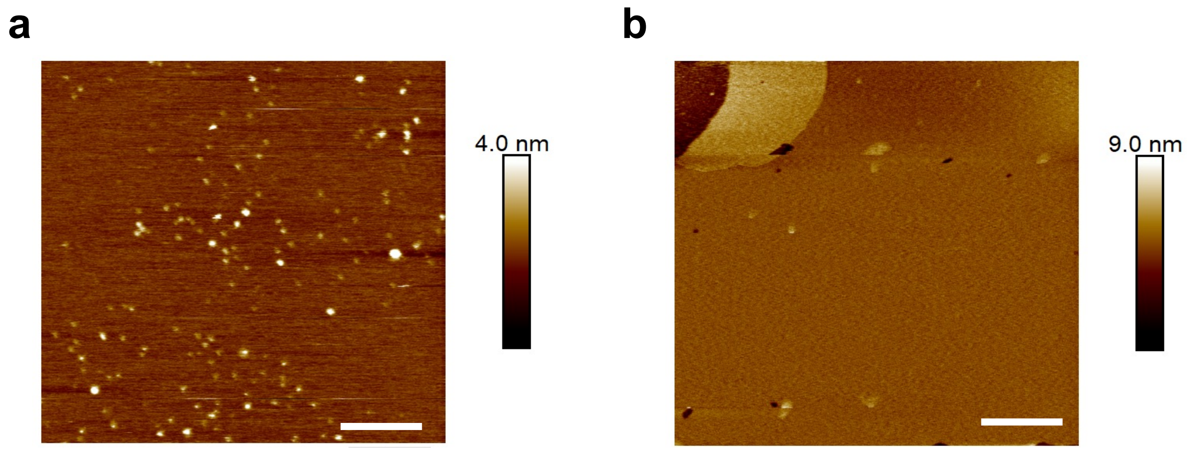

Figure S2. AFM images (2 x 2 µm) of supported lipid bilayers (SLBs) on mica. SLBs are prepared from (a) MsbA-WT-containing proteoliposomes or (b) empty liposomes. Height bars are shown to the right of each image. Scale bars are 400 nm.

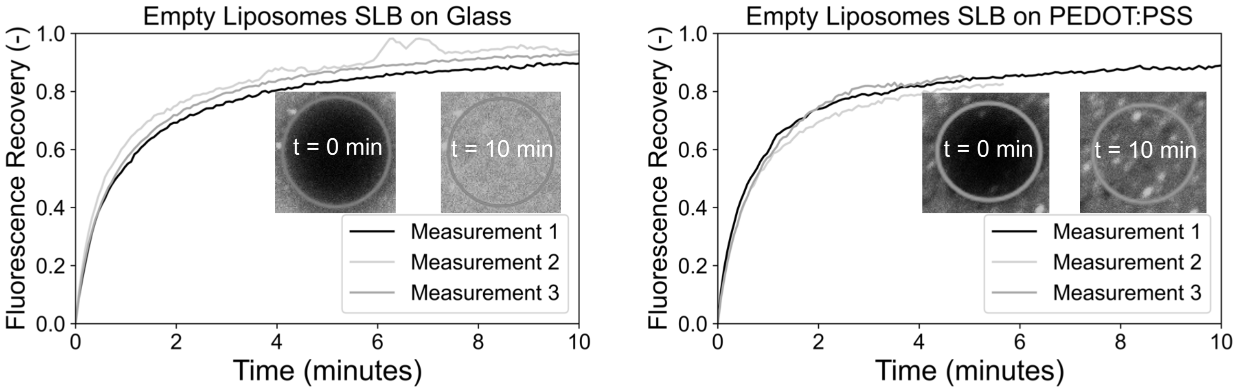

	Diffusion Coefficient ($\mu m^2/s$)	Mobile Fraction
Glass	1.22 ± 0.09	1.00 ± 0.01
PEDOT:PSS	1.31 ± 0.13	0.97 ± 0.02

Figure S3. FRAP characterisation on empty liposome SLBs. FRAP was conducted on (left) glass and (right) PEDOT:PSS. For both, the fluorescence in the bleached circle (diameter 30 μm) recovered over time. The calculated diffusion coefficient (D) and Mobile Fraction (MF) values on glass and PEDOT:PSS are shown in the table. Data was collected by bleaching three separate areas on the same bilayer. Error represents s.d. of the three measurements.

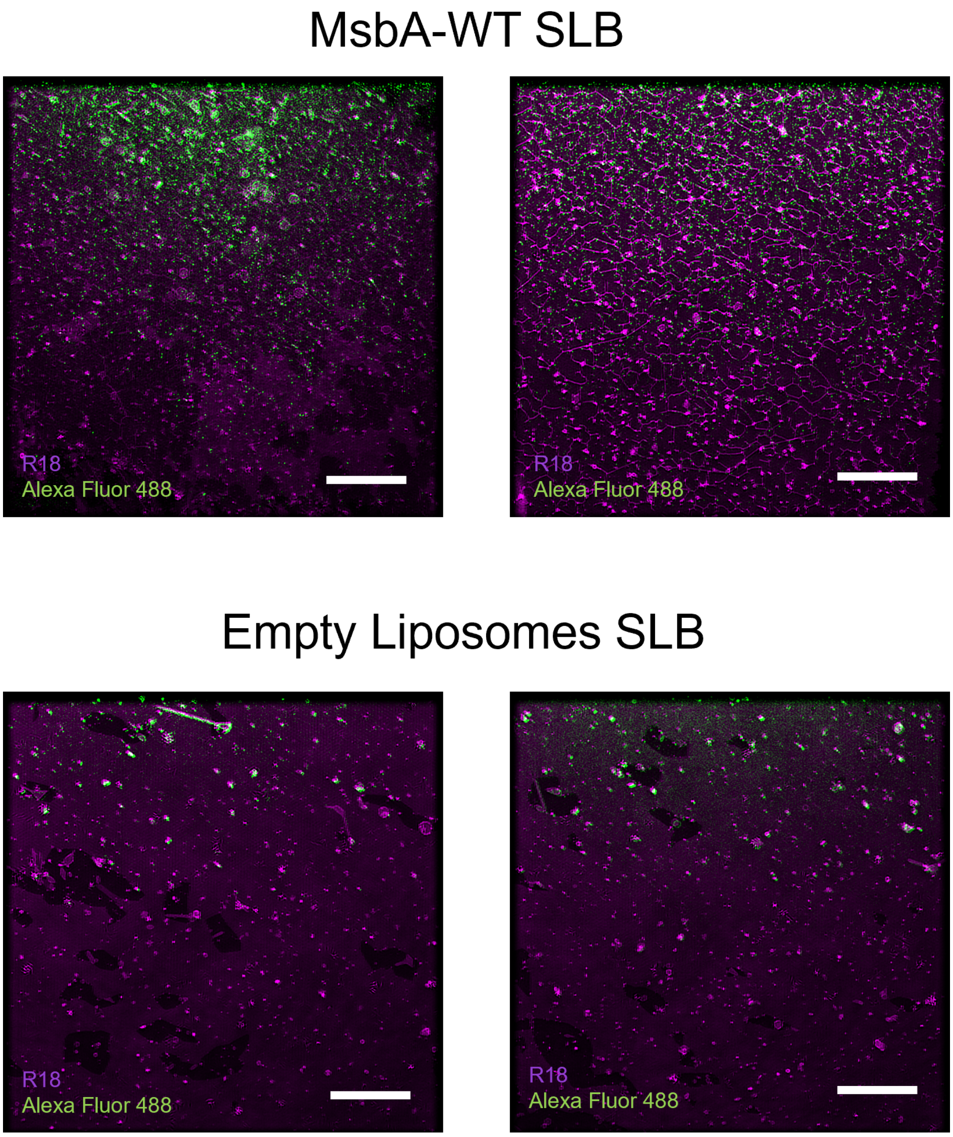

Figure S4. Immunofluorescence images for MsbA-WT-containing SLBs and SLBs prepared from empty liposomes on glass. Each bilayer is stained with R18 (purple) and then incubated with anti-His tag primary antibody and Alexa Fluor 488-labelled secondary antibody to detect the presence of MsbA-WT (green). The two images in each panel are taken from different areas of the same SLB. From these images, we show that SLBs can be formed on glass from both proteoliposomes and empty liposomes. The green fluorescence in the MsbA-WT SLB indicates the presence of MsbA protein. Scale bars are 10 μm.

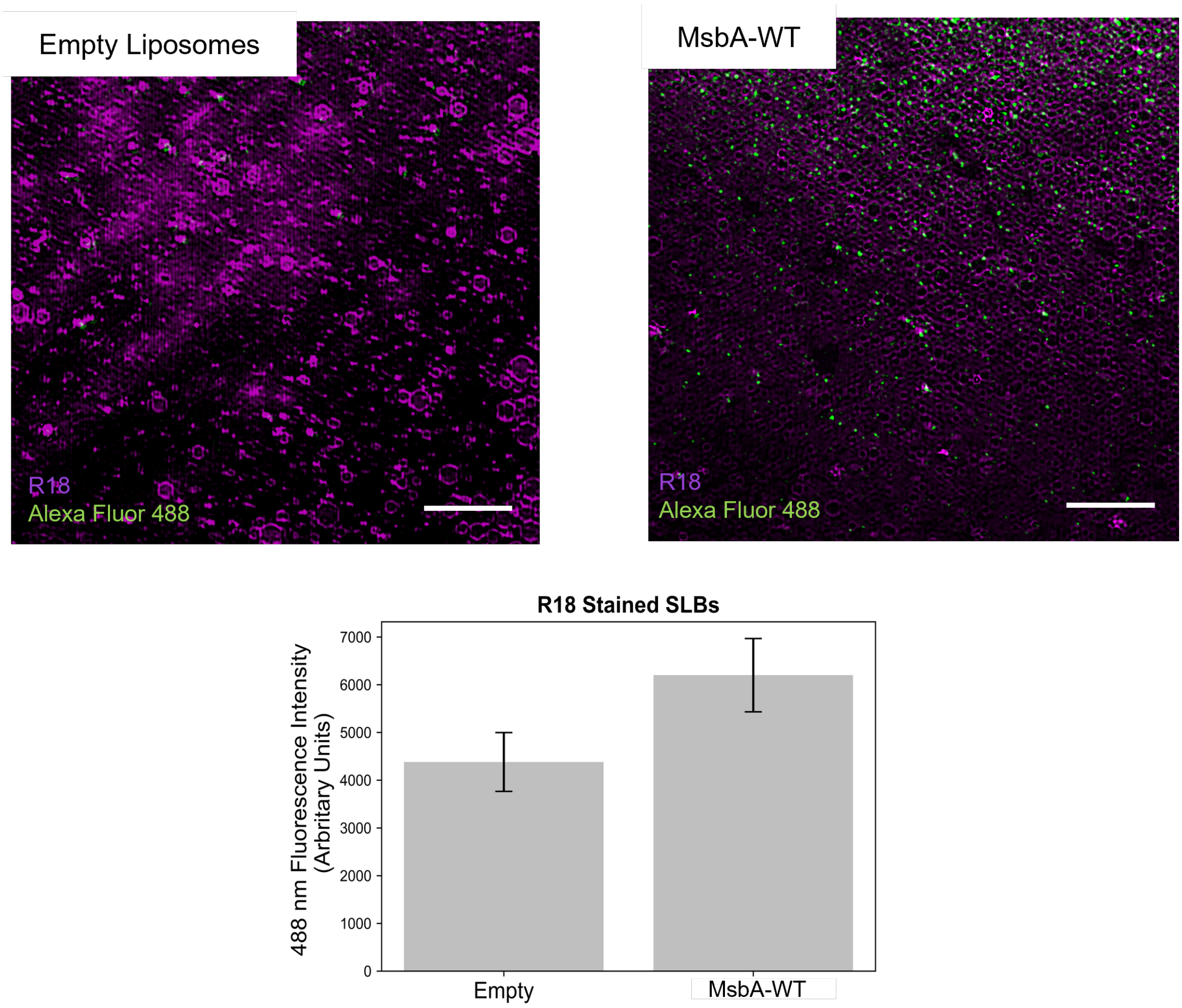

Figure S5. Immunofluorescence images for MsbA-WT-containing SLBs and empty SLBs on PEDOT:PSS. Each bilayer is stained with R18 (purple) and then incubated with anti-His tag primary antibody and Alexa Fluor 488-labelled secondary antibody to visualise the presence of MsbA-WT. The bar chart compares the 488 nm fluorescence between three separate images of the two types of SLBs. Error bars represent the standard deviation. Scale bars are 10 μm.

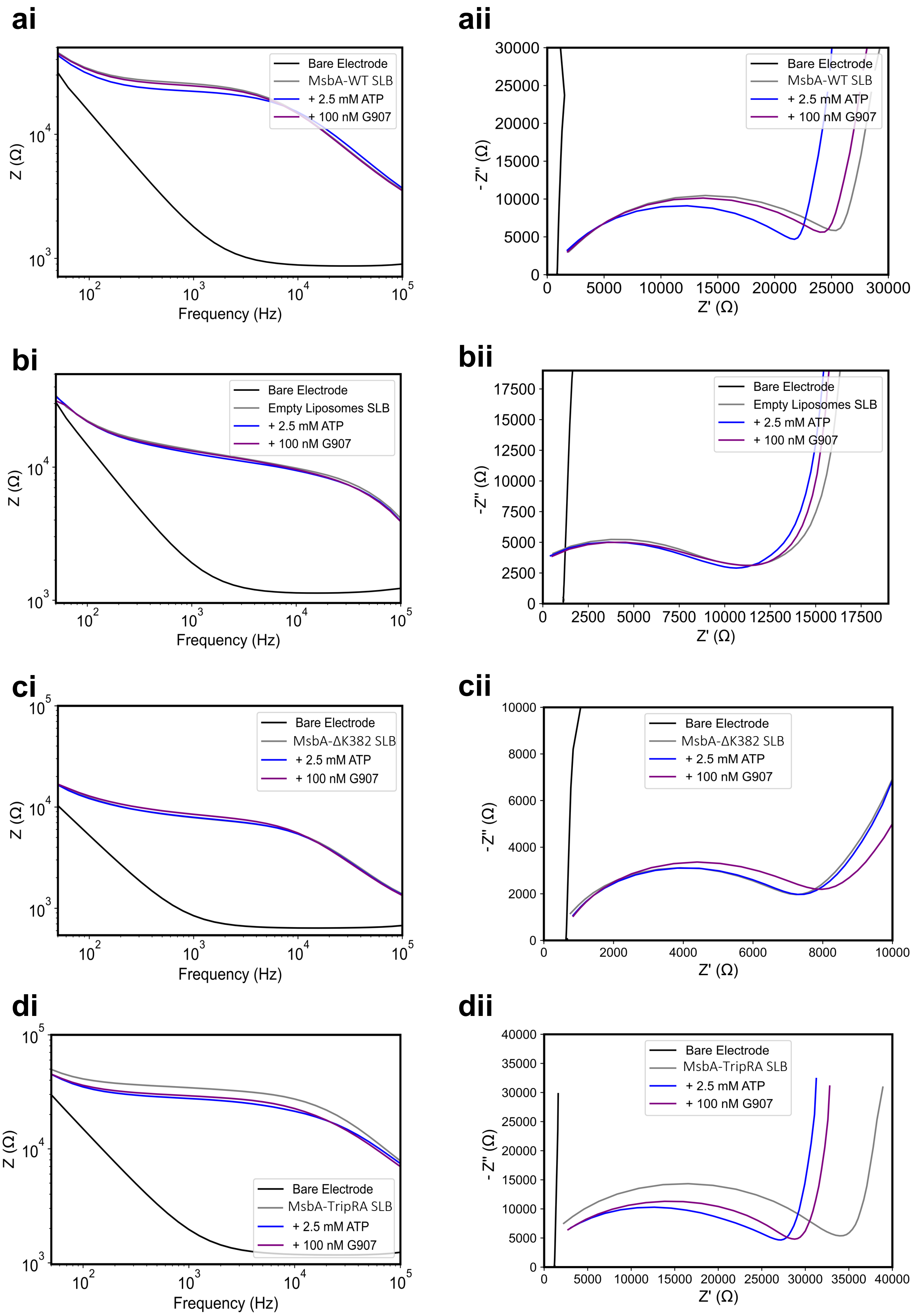

Figure S6. EIS measurements for the four SLB systems. (i) Bode and (ii) Nyquist plots for (a) MsbA-WT (n=3), (b) empty (n=3), (c) MsbA-ΔK382 (n=2), and (d) MsbA-TripRA (n=3) from which resistance changes were values were extracted. Presented here are example traces from a representative electrode from one SLB preparation for each (proteo)liposome condition. Z' and Z'' refer to the real and imaginary components of the impedance Z, respectively.

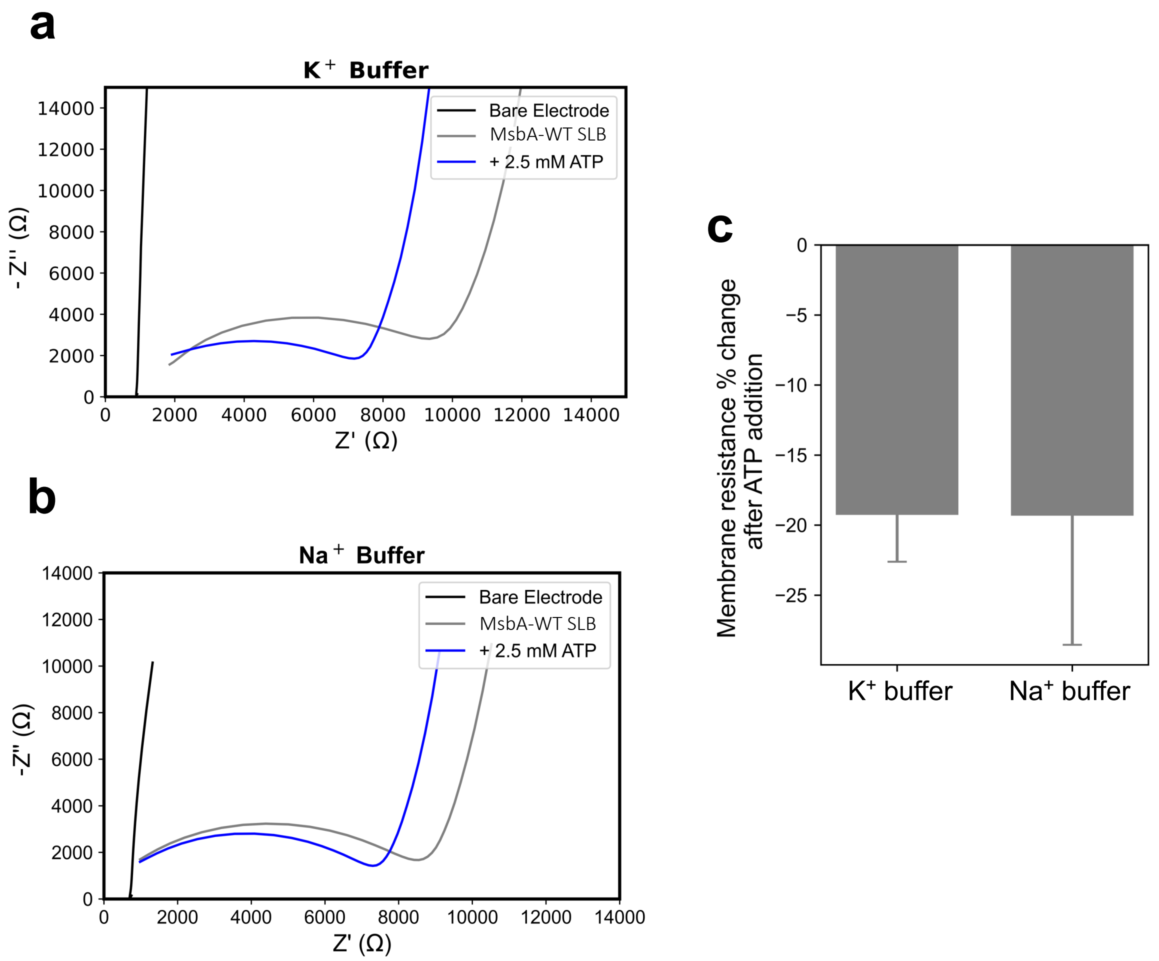

Figure S7. Ion permeation in MsbA-WT-containing SLBs before and after Mg-ATP addition. Nyquist plot for measurements in K^+ buffer (a) or Na^+ buffer (b). (c) Extracted resistance % change values for these experiments. Data represent three technical repeats for each condition with error bars representing standard deviation. These data show that the EIS responses for MsbA-WT SLBs in Na^+ buffer were not significantly different from those in K^+ buffer. Z' and Z'' refer to the real and imaginary components of the impedance Z, respectively.

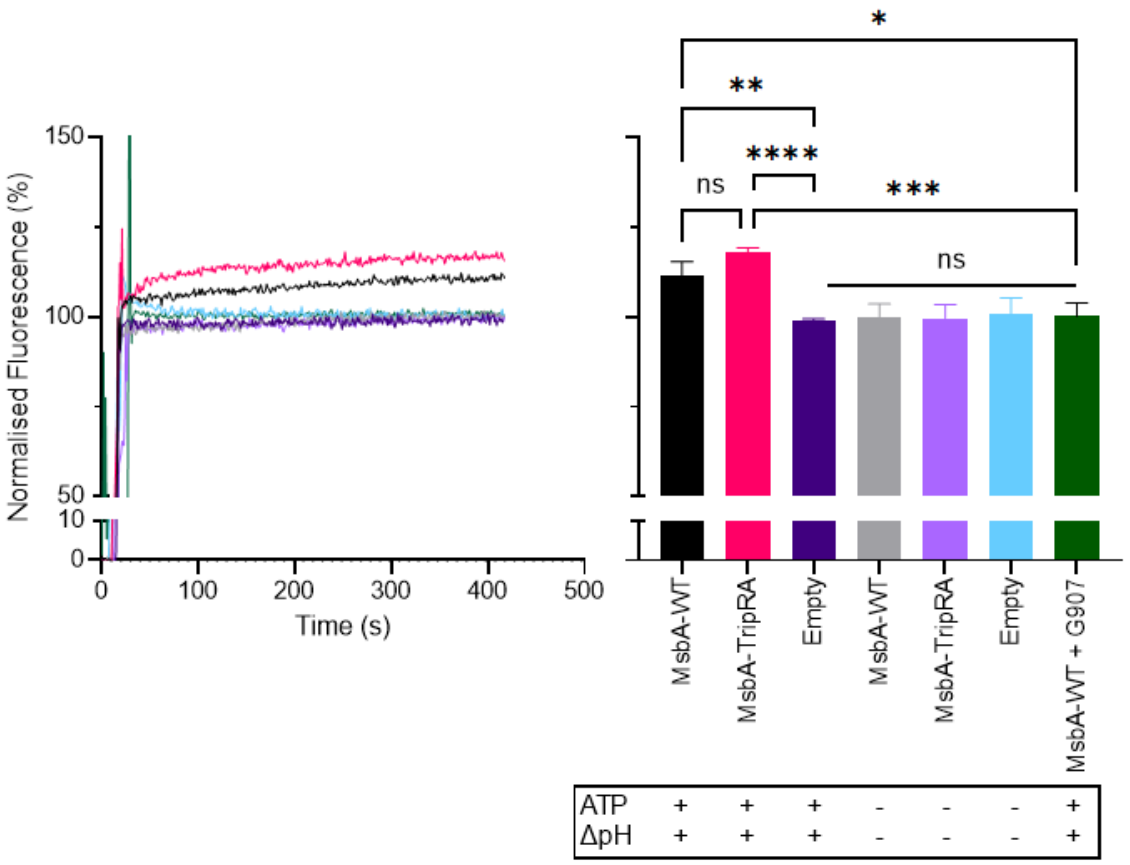

Figure S8. Ethidium transport into DNA-loaded proteoliposomes containing MsbA proteins in the 'NBD-out' orientation. Proteoliposomes were subjected to the presence of 2.5 mM Mg-ATP in the external buffer and the simultaneous imposition of a ΔpH (pH_{in} 6.8/pH_{out} 8.0) or were incubated without the ATP and ΔpH (pHin 6.8/pHout 6.8), after which 2 μM ethidium bromide was added to initiate transport. 100 nM of G907 (5x the reported in-vitro IC_{50}) was used to inhibit the transport activity of MsbA. Data represent observations in three or more independent experiments with independently prepared batches of proteoliposomes (n ≥ 2). Values in histograms show the significance of fluorescence levels at steady-state, and are expressed as mean ± s.e.m. (one-way analysis of variance; ns P>0.05; *P≤0.05; **P≤0.01; ***P≤0.001; ****P≤0.0001). These data show that our proteoliposome preparations for MsbA-WT and MsbA-TripRA are transport-active in the ethidium transport measurements.

Table S1. Resistance values extracted from EIS data for the four SLB systems in Figure S6. (a) MsbA-WT (n=3), (b) empty (n=3), (c) MsbA-ΔK382 (n=2), and (d) MsbA-TripRA (n=3). Data represent mean from separate biological repeats prior to correcting for electrode variation.

a

	Resistance ($\Omega.cm^2$)	
	**mean**	**s.e.m.**
Liposome buffer	24.89	3.24
MsbA-WT SLB	216.74	37.00
+ ATP	173.02	31.50
+ G907	199.37	37.92

b

	Resistance ($\Omega.cm^2$)	
	**mean**	**s.e.m.**
Liposome buffer	21.71	0.63
Empty liposomes SLB	200.28	48.41
+ ATP	184.52	40.28
+ G907	187.09	39.66

c

	Resistance ($\Omega.cm^2$)	
	**mean**	**s.e.m.**
Liposome buffer	24.02	0.58
MsbA-ΔK382 SLB	104.23	26.06
+ ATP	100.28	26.67
+ G907	108.75	28.38

d

	Resistance ($\Omega.cm^2$)	
	**mean**	**s.e.m.**
Liposome buffer	24.61	0.64
MsbA-TripRA SLB	203.33	59.34
+ ATP	156.19	46.04
+ G907	170.99	50.02
